# The diagnostic challenge of myocardial infarction in critically ill patients: do high-sensitivity troponin measurements add more clarity or more confusion?

**DOI:** 10.1186/cc13909

**Published:** 2014-06-05

**Authors:** Kada Klouche, Olivier Jonquet, Jean Paul Cristol

**Affiliations:** 1 Intensive Care Medicine Department, University of Montpellier, Lapeyronie Hospital, 371, Av Doyen G. Giraud, Montpellier 34295, France; 2 U 1046, University of Montpellier, 1, Arnaud de Villeneuve, 371, Av Doyen G. Giraud, Montpellier 34295, France; 3 Biochemistry Department, Lapeyronie University Hospital, 371, Av Doyen G. Giraud, Montpellier 34295, France

## Abstract

In ICU settings, the diagnosis and treatment of acute myocardial infarction (AMI) are challenging, partly because cardiac troponin increase occurs frequently. In the previous issue of *Critical Care*, Ostermann and colleagues reported that myocardial infarction (MI), screened by plasma troponin and electrocardiography changes, is common and often clinically unrecognized in the ICU. Although the clinical significance of underdiagnosed MIs remains unclear, this approach may help to target and further investigate the at-risk population for appropriate therapy.

## 

In the previous issue of *Critical Care*, Ostermann and colleagues [1] studied the impact of serial troponin measurements on the detection and diagnosis of acute myocardial infarction (AMI) in patients in the ICU. AMI indeed represents a specific diagnostic and therapeutic challenge in ICU settings. Its universal definition - rise or fall (or both) of cardiac biomarkers, preferably cardiac troponin (cTn), above the 99th percentile of the upper reference limit associated with clinical, electrical, or imaging symptoms of ischemia [2] - has limitations in the ICU. Sedation and analgesia mask ischemic chest pain, many of the accompanying signs of (MI) such as hypotension and arrhythmias are often non-specific, and cardiac biomarkers are somewhat difficult to interpret. In addition, imaging investigations to assess MI are not routinely performed and are initiated only when the diagnosis is strongly suspected. Nevertheless, identifying patients with ischemia and infarction is important from diagnostic, prognostic, and treatment perspectives.

Intensivists frequently rely on electrocardiography (ECG) evidence of myocardial ischemia, but continuous 2- or 12-lead ECG routine screening remains poorly satisfactory because of a low sensitivity and lack of specificity compared with non-ICU patients [3,4]. It may be improved when associated with knowledge of cTn values [4], but the incidence of raised serum cTn is high in critically ill patients, varying from 32% to 53% [5]. Interestingly, most of these patients (nearly 70%) do not have coronary diseases as assessed by stress echocardiography or post-mortem diagnosis [6]. In the prospective observational study by Ostermann and colleagues [1], 144 consecutive patients admitted to the ICU for non-cardiac reasons were screened by recording ECGs and plasma high-sensitivity cardiac troponin T (hs-cTnT). (Results were blinded for the medical team caring for the patient if not ordered on clinical grounds.) Among patients studied, 121 (84%) experienced at least one plasma hs-cTnT of more than 15 ng/L and only 7 (6%) had a normal value at admission. After analyses, they were classified into four groups: (a) definite MI: cardiac troponin T (cTnT) of at least 15 ng/L and ECG changes of MI: 20 (14%); (b) possible MI: cTnT of at least 15 ng/L and ischemic ECG changes: 39 (27%); (c) troponin rise alone (cTnT of at least 15 ng/L): 62 (43%); or (d) normal: 23 (14%). More than 40% of patients (59) had study-identified MIs, but only 12 of these were suspected by the ICU team, meaning that more than 80% of MIs were misdiagnosed. However, a similar mortality was observed between clinically recognized and unrecognized MIs with a longer length of ICU stay in the former (17 versus 7 ICU days and 51 versus 18 hospital days, *P* = 0.02).

These findings confirm previous reports underlining that MI, routinely screened by combined ECG changes and plasma hs-cTnT, is quite common and underdiagnosed in ICUs [7]. It is, however, unclear whether knowledge of cTn values when interpreting ECGs leads to an appropriate diagnosis or over-diagnosis of AMI. Indeed, clinical significance of MI remains questionable since Ostermann and colleagues [1] did observe a similar mortality whether it was clinically recognized or not.

ICU patients are at high risk for myocardial ischemia because of older age, increased intrinsic and extrinsic sympathetic stimulation, hypoxia, vasopressor use, and coagulation disorders. However, myocardial necrosis may be induced by coronary atherothrombosis or by other triggers that cause an imbalance between coronary oxygen supply and myocardial oxygen demand [8]. The presence of elevated cTn, in addition to ECG changes, may help to make a decision to rule in or out MI or to ascertain it as did Ostermann and colleagues [1]. The recent analytical methods using high-sensitivity cardiac troponin (hs-cTn) assays are far more sensitive and improve the diagnosis of MI but at the price of a reduction in specificity [9].

Thus, several issues regarding the use of hs-cTn in the ICU should be addressed. First, determination of the 99th percentile of hs-cTn could be either variable in this specific population mostly characterized by older age and comorbidities. While this value increases in older (more than 65 years old) patients admitted to the emergency room [10], it dramatically decreases when, in order to screen volunteers, questionnaires for comorbidity, glomerular filtration rate level, ECG, or cardiac imaging were added [11]. Therefore, the universal upper reference limit lacks specificity in the critically ill. Second, the appreciation of rise or fall of cTn levels during a 3- or 6-hour kinetic, though still debated, seems to be determinant to rule in AMI [11,12]. In the ICU, the search for the optimal delta change should take into account the patient’s specific conditions such as vasopressor-induced troponin rise [11]. Finally, an increase in cTn is due not only to myocardial cell necrosis but also to the release of cytosolic troponin pool from damaged myocytes that could be detected in plasma with high-sensitivity assay in both reversible and irreversible myocardial injury. Situations leading to high-sensitivity troponin level rise (recently reviewed in [12,13]) are particularly frequent in the ICU and include kidney disease, heart or respiratory failure (or both), sepsis, pulmonary embolism, stroke, Tako-tsubo, myocarditis, trauma, cardiopulmonary resuscitation, and tachycardia.

In conclusion, the diagnosis of AMI remains challenging in the ICU, and the use of high-sensitivity troponin T as a diagnostic tool should be prudent because it may lead to an overestimation of evidence of ischemia. To increase the accuracy for diagnosing MI, one should reserve invasive or non-invasive procedures, such as bedside echocardiography or coronary angiography, to a targeted population. The work of Ostermann and colleagues [1] provides further information to best identify this at-risk population. Yet the equivalence in outcomes between clinically recognized and unrecognized MI may reflect the challenge of appropriate and timely medical intervention for MIs. Larger trials will be necessary to further evaluate the utility of troponin-based clinical algorithms to an improvement in outcomes.

## Abbreviations

AMI: Acute myocardial infarction; cTn: Cardiac troponin; cTnT: Cardiac troponin T; ECG: Electrocardiography; hs-cTn: High-sensitivity cardiac troponin; hs-cTnT: High-sensitivity cardiac troponin T; MI: Myocardial infarction.

## Competing interests

The authors declare that they have no competing interests.

## Authors’ contributions

All authors read and approved the final manuscript.
